# 3,5-Bis(4-hy­droxy­phen­yl)-4*H*-1,2,4-triazol-4-amine monohydrate

**DOI:** 10.1107/S1600536810032915

**Published:** 2010-08-28

**Authors:** Sidik Silong, Mohamad Zaki Ab. Rahman, Mansor Hj Ahmad, Huey Chong Kwong, Seik Weng Ng

**Affiliations:** a Department of Chemistry, Universiti Putra Malaysia, 43400 Serdang, Malaysia; b Department of Chemistry, University of Malaya, 50603 Kuala Lumpur, Malaysia

## Abstract

The triazole ring in the title compound, C_14_H_12_N_4_O_2_·H_2_O, makes dihedral angles of 36.9 (1) and 37.3 (1)° with the two benzene rings. Each hy­droxy group is a hydrogen-bond donor to a two-coordinate N atom of an adjacent mol­ecule; these O—H⋯N hydrogen bonds generate a layer parallel to the *ab* plane. Adjacent layers are linked by N—-H⋯O and O_water_—H⋯O hydrogen bonds into a three-dimensional network.

## Related literature

For two modifications of 4-amino-3,5-diphenyl-1,2,4-triazole, see: Ikemi *et al.* (2002 ▶); Zhang *et al.* (2009 ▶). For comparison structures, see: Wang *et al.* (2006 ▶); Zachara *et al.* (2004 ▶); Bentiss *et al.* (1998 ▶).
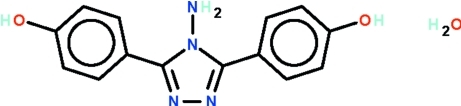


## Experimental

### 

#### Crystal data

C_14_H_12_N_4_O_2_·H_2_O
*M* *_r_* = 286.29
Orthorhombic, 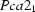

*a* = 10.6659 (5) Å
*b* = 15.9790 (8) Å
*c* = 7.4632 (4) Å
*V* = 1271.96 (11) Å^3^
*Z* = 4
Cu *K*α radiation
μ = 0.90 mm^−1^
*T* = 100 K
0.30 × 0.10 × 0.05 mm

#### Data collection

Oxford Diffraction Gemini E diffractometer
Absorption correction: multi-scan (*CrysAlis PRO*; Oxford Diffraction, 2010 ▶) *T*_min_ = 0.773, *T*_max_ = 0.956
2460 measured reflections
1275 independent reflections
1172 reflections with *I* > 2σ(*I*)
*R*_int_ = 0.019

#### Refinement

*R*[*F* ^2^ > 2σ(*F* ^2^)] = 0.034
*wR*(*F* ^2^) = 0.097
*S* = 1.05
1275 reflections
215 parameters
8 restraints
H atoms treated by a mixture of independent and constrained refinement
Δρ_max_ = 0.23 e Å^−3^
Δρ_min_ = −0.24 e Å^−3^

### 

Data collection: *CrysAlis PRO* (Oxford Diffraction, 2010 ▶); cell refinement: *CrysAlis PRO*; data reduction: *CrysAlis PRO*; program(s) used to solve structure: *SHELXS97* (Sheldrick, 2008 ▶); program(s) used to refine structure: *SHELXL97* (Sheldrick, 2008 ▶); molecular graphics: *X-SEED* (Barbour, 2001 ▶); software used to prepare material for publication: *publCIF* (Westrip, 2010 ▶).

## Supplementary Material

Crystal structure: contains datablocks global, I. DOI: 10.1107/S1600536810032915/bt5326sup1.cif

Structure factors: contains datablocks I. DOI: 10.1107/S1600536810032915/bt5326Isup2.hkl

Additional supplementary materials:  crystallographic information; 3D view; checkCIF report

## Figures and Tables

**Table 1:** Hydrogen-bond geometry (Å, °)

*D*—H⋯*A*	*D*—H	H⋯*A*	*D*⋯*A*	*D*—H⋯*A*
O1—H1o⋯N3^i^	0.84 (3)	1.92 (3)	2.730 (2)	163 (3)
O2—H2o⋯N4^ii^	0.84 (3)	2.02 (3)	2.850 (2)	168 (4)
O1w—H11⋯O2^iii^	0.85 (3)	2.19 (3)	3.020 (3)	166 (4)
N1—H1n⋯O1w	0.87 (3)	2.08 (3)	2.943 (4)	173 (4)
N1—H2n⋯O1^iv^	0.86 (3)	2.36 (3)	3.202 (4)	164 (3)

Symmetry codes: (i) 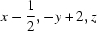; (ii) 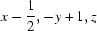; (iii) 
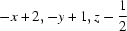
; (iv) 
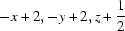
.

**Fig. 1.**
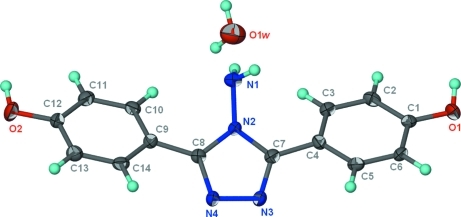
Anisotropic displacement ellipsoid plot (Barbour, 2001) of C14H12N4O2.H2O at the 70% probability level; hydrogen atoms are drawn as spheres of arbitrary radius.

## supplementary crystallographic information

### Comment

The class of 3,5-bis(*n*-hydroxyphenyl)-4-amino-1,2,4-triazoles represents a class of efficient corrosion inhibitors that are synthesized from *n*-hydroxybenzonitrile, hydrazine and hydrazine sulfate. The pure product is obtained in a number of steps that involve acidification/basification followed by recrystallization, as exemplified by 3,5-bis(4-hydroxyphenyl)-4-amino-1,2,4-triazole (Bentiss *et al.*, 1998). The compound can be synthesized, more conveniently, though a microwave route. The compound is, in fact, a monohydrate (Scheme I, Fig. 1).

### Experimental

4-Hydroxybenzonitrile (3 mmol), hydrazine dihydrochloride (1 mmol) and anhydrous hydrazine (3 mmol) along with *n*-butanol (3 ml) were placed in a microwave synthesizer tube. The tube was irradiated in a CEM Discovery Synthesizer. The magnetron was set to 'normal' and the temperature to 403 K. The tube was irradiated for 8 minutes. Water (10 ml) was added to dissolve the contents. The solution was set aside for the growth of the faint-yellow crystals, which separated after 3 days.

### Refinement

Carbon-bound H-atoms were placed in calculated positions (C—H 0.95 Å) and were included in the refinement in the riding model approximation, with *U*(H) set to 1.2*U*(C). The amino and water H-atoms were located in a difference Fourier map, and were refined with distance restraints [N–H 0.86 + 0.01 Å and O–H 0.84 + 0.01 Å]; their displacement parameters were freely refined. For the water molecule, the H···H distance was restrained to 1.37±0.01 Å. 402 Friedel pairs were merged.

### Crystal data

**Table:**

C_14_H_12_N_4_O_2_·H_2_O	*F*(000) = 600
*M**_r_* = 286.29	*D*_x_ = 1.495 Mg m^−^^3^
Orthorhombic, *P**c**a*2_1_	Cu *K*α radiation, λ = 1.54178 Å
Hall symbol: P 2c -2ac	Cell parameters from 1690 reflections
*a* = 10.6659 (5) Å	θ = 4.1–70.4°
*b* = 15.9790 (8) Å	µ = 0.90 mm^−^^1^
*c* = 7.4632 (4) Å	*T* = 100 K
*V* = 1271.96 (11) Å^3^	Prism, yellow
*Z* = 4	0.30 × 0.10 × 0.05 mm

### Data collection

**Table:**

Oxford Diffraction Gemini E diffractometer	1275 independent reflections
Radiation source: fine-focus sealed tube	1172 reflections with *I* > 2σ(*I*)
graphite	*R*_int_ = 0.019
Detector resolution: 16.1952 pixels mm^-1^	θ_max_ = 70.0°, θ_min_ = 5.0°
ω scans	*h* = −11→12
Absorption correction: multi-scan (*CrysAlis PRO*; Oxford Diffraction, 2010)	*k* = −14→19
*T*_min_ = 0.773, *T*_max_ = 0.956	*l* = −9→8
2460 measured reflections	

### Refinement

**Table:**

Refinement on *F*^2^	Secondary atom site location: difference Fourier map
Least-squares matrix: full	Hydrogen site location: inferred from neighbouring sites
*R*[*F*^2^ > 2σ(*F*^2^)] = 0.034	H atoms treated by a mixture of independent and constrained refinement
*wR*(*F*^2^) = 0.097	*w* = 1/[σ^2^(*F*_o_^2^) + (0.0762*P*)^2^ + 0.0334*P*] where *P* = (*F*_o_^2^ + 2*F*_c_^2^)/3
*S* = 1.05	(Δ/σ)_max_ = 0.001
1275 reflections	Δρ_max_ = 0.23 e Å^−^^3^
215 parameters	Δρ_min_ = −0.24 e Å^−^^3^
8 restraints	Extinction correction: *SHELXL97* (Sheldrick, 2008), Fc^*^=kFc[1+0.001xFc^2^λ^3^/sin(2θ)]^-1/4^
Primary atom site location: structure-invariant direct methods	Extinction coefficient: 0.0033 (6)

### Fractional atomic coordinates and isotropic or equivalent isotropic displacement parameters (Å^2^)

**Table:**

	*x*	*y*	*z*	*U*_iso_*/*U*_eq_	
O1	1.05710 (16)	1.15554 (10)	0.5001 (3)	0.0257 (5)	
O2	1.07428 (16)	0.33922 (9)	0.5626 (3)	0.0252 (4)	
O1W	0.8047 (2)	0.76244 (13)	0.3567 (3)	0.0375 (5)	
C1	1.0874 (2)	1.07317 (13)	0.5123 (3)	0.0175 (5)	
C2	1.0093 (2)	1.01053 (14)	0.4426 (3)	0.0184 (5)	
H2	0.9335	1.0254	0.3839	0.022*	
C3	1.0424 (2)	0.92705 (14)	0.4592 (3)	0.0175 (5)	
H3	0.9886	0.8848	0.4135	0.021*	
C4	1.1546 (2)	0.90481 (12)	0.5428 (3)	0.0157 (5)	
C5	1.2323 (2)	0.96757 (13)	0.6105 (3)	0.0166 (5)	
H5	1.3087	0.9528	0.6676	0.020*	
C6	1.1993 (2)	1.05105 (13)	0.5954 (4)	0.0173 (5)	
H6	1.2531	1.0932	0.6418	0.021*	
C7	1.19711 (19)	0.81723 (13)	0.5495 (4)	0.0165 (5)	
C8	1.20070 (19)	0.68071 (13)	0.5660 (3)	0.0154 (5)	
C9	1.1602 (2)	0.59292 (12)	0.5747 (3)	0.0156 (5)	
C10	1.0488 (2)	0.56628 (14)	0.4941 (3)	0.0166 (5)	
H10	0.9928	0.6063	0.4444	0.020*	
C11	1.0195 (2)	0.48188 (13)	0.4864 (3)	0.0177 (5)	
H11A	0.9448	0.4640	0.4288	0.021*	
C12	1.0998 (2)	0.42316 (13)	0.5633 (4)	0.0176 (5)	
C13	1.2108 (2)	0.44890 (13)	0.6442 (3)	0.0187 (5)	
H13	1.2658	0.4089	0.6960	0.022*	
C14	1.2406 (2)	0.53350 (13)	0.6487 (3)	0.0165 (5)	
H14	1.3167	0.5511	0.7029	0.020*	
N1	0.9975 (2)	0.74477 (11)	0.6363 (4)	0.0212 (5)	
N2	1.1228 (2)	0.74854 (9)	0.5740 (3)	0.0161 (5)	
N3	1.31389 (18)	0.79302 (10)	0.5291 (3)	0.0185 (5)	
N4	1.31642 (18)	0.70604 (10)	0.5380 (3)	0.0188 (5)	
H1O	0.9790 (11)	1.161 (2)	0.508 (5)	0.042 (10)*	
H2O	0.9976 (14)	0.331 (3)	0.542 (7)	0.082 (16)*	
H11	0.827 (3)	0.7358 (19)	0.264 (3)	0.053 (12)*	
H12	0.7286 (15)	0.7500 (18)	0.376 (6)	0.072 (17)*	
H1N	0.945 (3)	0.7523 (17)	0.549 (4)	0.046 (11)*	
H2N	0.987 (4)	0.7803 (18)	0.722 (4)	0.055 (11)*	

### Atomic displacement parameters (Å^2^)

**Table:**

	*U*^11^	*U*^22^	*U*^33^	*U*^12^	*U*^13^	*U*^23^
O1	0.0173 (8)	0.0142 (7)	0.0455 (13)	0.0051 (6)	0.0026 (8)	0.0015 (8)
O2	0.0207 (8)	0.0137 (7)	0.0412 (12)	−0.0040 (6)	−0.0055 (9)	0.0015 (8)
O1W	0.0296 (11)	0.0460 (11)	0.0368 (13)	−0.0008 (9)	0.0020 (9)	−0.0085 (11)
C1	0.0144 (11)	0.0154 (10)	0.0226 (14)	0.0001 (8)	0.0072 (10)	0.0021 (9)
C2	0.0144 (10)	0.0202 (11)	0.0207 (13)	0.0038 (8)	−0.0007 (9)	0.0006 (10)
C3	0.0147 (11)	0.0166 (10)	0.0212 (13)	−0.0018 (8)	0.0015 (9)	−0.0003 (9)
C4	0.0166 (10)	0.0124 (10)	0.0181 (11)	0.0013 (8)	0.0030 (10)	−0.0009 (9)
C5	0.0129 (10)	0.0187 (10)	0.0182 (12)	−0.0002 (8)	0.0013 (9)	0.0012 (9)
C6	0.0147 (10)	0.0144 (10)	0.0229 (14)	−0.0039 (8)	0.0033 (9)	−0.0023 (10)
C7	0.0154 (10)	0.0154 (10)	0.0186 (13)	−0.0014 (8)	0.0011 (10)	−0.0010 (10)
C8	0.0149 (10)	0.0148 (10)	0.0164 (12)	0.0001 (8)	0.0006 (10)	0.0001 (10)
C9	0.0157 (10)	0.0133 (10)	0.0177 (12)	−0.0008 (8)	0.0025 (10)	−0.0002 (9)
C10	0.0136 (11)	0.0170 (10)	0.0192 (13)	0.0021 (8)	0.0014 (9)	0.0010 (9)
C11	0.0159 (10)	0.0185 (12)	0.0187 (12)	−0.0021 (8)	0.0010 (10)	−0.0004 (10)
C12	0.0158 (11)	0.0142 (9)	0.0229 (12)	−0.0018 (8)	0.0026 (10)	−0.0037 (10)
C13	0.0168 (11)	0.0166 (11)	0.0225 (14)	0.0019 (8)	0.0006 (10)	0.0038 (9)
C14	0.0114 (9)	0.0197 (9)	0.0184 (13)	−0.0007 (9)	0.0004 (9)	−0.0018 (9)
N1	0.0135 (11)	0.0214 (10)	0.0288 (12)	−0.0006 (7)	0.0036 (10)	−0.0013 (9)
N2	0.0129 (9)	0.0121 (9)	0.0234 (11)	−0.0013 (6)	0.0015 (9)	0.0002 (7)
N3	0.0144 (9)	0.0118 (9)	0.0294 (13)	−0.0011 (6)	−0.0004 (8)	0.0010 (9)
N4	0.0148 (9)	0.0108 (9)	0.0307 (13)	−0.0007 (6)	0.0001 (9)	−0.0008 (9)

### Geometric parameters (Å, °)

**Table:**

O1—C1	1.359 (3)	C7—N2	1.366 (3)
O1—H1O	0.84 (3)	C8—N4	1.316 (3)
O2—C12	1.369 (2)	C8—N2	1.367 (3)
O2—H2O	0.84 (3)	C8—C9	1.469 (3)
O1w—H11	0.85 (3)	C9—C14	1.393 (3)
O1w—H12	0.85 (3)	C9—C10	1.399 (3)
C1—C6	1.391 (3)	C10—C11	1.385 (3)
C1—C2	1.402 (3)	C10—H10	0.9500
C2—C3	1.386 (3)	C11—C12	1.393 (3)
C2—H2	0.9500	C11—H11A	0.9500
C3—C4	1.395 (3)	C12—C13	1.392 (3)
C3—H3	0.9500	C13—C14	1.389 (3)
C4—C5	1.396 (3)	C13—H13	0.9500
C4—C7	1.472 (3)	C14—H14	0.9500
C5—C6	1.384 (3)	N1—N2	1.416 (3)
C5—H5	0.9500	N1—H1N	0.87 (3)
C6—H6	0.9500	N1—H2N	0.86 (3)
C7—N3	1.313 (3)	N3—N4	1.392 (3)
			
C1—O1—H1O	109 (2)	C14—C9—C10	119.06 (19)
C12—O2—H2O	110 (3)	C14—C9—C8	119.2 (2)
H11—O1W—H12	106.8 (17)	C10—C9—C8	121.4 (2)
O1—C1—C6	118.7 (2)	C11—C10—C9	120.4 (2)
O1—C1—C2	121.7 (2)	C11—C10—H10	119.8
C6—C1—C2	119.60 (19)	C9—C10—H10	119.8
C3—C2—C1	120.2 (2)	C10—C11—C12	120.0 (2)
C3—C2—H2	119.9	C10—C11—H11A	120.0
C1—C2—H2	119.9	C12—C11—H11A	120.0
C2—C3—C4	120.2 (2)	O2—C12—C13	117.4 (2)
C2—C3—H3	119.9	O2—C12—C11	122.4 (2)
C4—C3—H3	119.9	C13—C12—C11	120.15 (19)
C3—C4—C5	119.21 (19)	C14—C13—C12	119.5 (2)
C3—C4—C7	121.4 (2)	C14—C13—H13	120.2
C5—C4—C7	119.2 (2)	C12—C13—H13	120.2
C6—C5—C4	120.8 (2)	C13—C14—C9	120.9 (2)
C6—C5—H5	119.6	C13—C14—H14	119.6
C4—C5—H5	119.6	C9—C14—H14	119.6
C5—C6—C1	119.9 (2)	N2—N1—H1N	111 (3)
C5—C6—H6	120.0	N2—N1—H2N	110 (3)
C1—C6—H6	120.0	H1N—N1—H2N	112 (3)
N3—C7—N2	109.22 (19)	C7—N2—C8	106.2 (2)
N3—C7—C4	124.7 (2)	C7—N2—N1	128.73 (17)
N2—C7—C4	126.12 (19)	C8—N2—N1	123.65 (17)
N4—C8—N2	109.47 (19)	C7—N3—N4	107.88 (16)
N4—C8—C9	125.19 (19)	C8—N4—N3	107.25 (16)
N2—C8—C9	125.21 (19)		
			
O1—C1—C2—C3	−179.3 (2)	C10—C11—C12—O2	178.7 (2)
C6—C1—C2—C3	1.1 (4)	C10—C11—C12—C13	−1.5 (4)
C1—C2—C3—C4	−1.0 (4)	O2—C12—C13—C14	−179.8 (2)
C2—C3—C4—C5	0.5 (4)	C11—C12—C13—C14	0.4 (4)
C2—C3—C4—C7	−175.0 (2)	C12—C13—C14—C9	0.6 (4)
C3—C4—C5—C6	0.0 (4)	C10—C9—C14—C13	−0.4 (3)
C7—C4—C5—C6	175.5 (2)	C8—C9—C14—C13	−174.0 (2)
C4—C5—C6—C1	0.1 (4)	N3—C7—N2—C8	−0.5 (3)
O1—C1—C6—C5	179.7 (2)	C4—C7—N2—C8	178.4 (2)
C2—C1—C6—C5	−0.6 (3)	N3—C7—N2—N1	166.0 (2)
C3—C4—C7—N3	140.3 (3)	C4—C7—N2—N1	−15.2 (4)
C5—C4—C7—N3	−35.2 (4)	N4—C8—N2—C7	−0.1 (3)
C3—C4—C7—N2	−38.4 (4)	C9—C8—N2—C7	−176.1 (2)
C5—C4—C7—N2	146.2 (3)	N4—C8—N2—N1	−167.4 (2)
N4—C8—C9—C14	34.9 (4)	C9—C8—N2—N1	16.6 (4)
N2—C8—C9—C14	−149.7 (2)	N2—C7—N3—N4	0.8 (3)
N4—C8—C9—C10	−138.5 (3)	C4—C7—N3—N4	−178.0 (2)
N2—C8—C9—C10	36.9 (4)	N2—C8—N4—N3	0.5 (3)
C14—C9—C10—C11	−0.7 (4)	C9—C8—N4—N3	176.6 (2)
C8—C9—C10—C11	172.7 (2)	C7—N3—N4—C8	−0.8 (2)
C9—C10—C11—C12	1.7 (4)		

### Hydrogen-bond geometry (Å, °)

**Table:**

*D*—H···*A*	*D*—H	H···*A*	*D*···*A*	*D*—H···*A*
O1—H1o···N3^i^	0.84 (3)	1.92 (3)	2.730 (2)	163 (3)
O2—H2o···N4^ii^	0.84 (3)	2.02 (3)	2.850 (2)	168 (4)
O1w—H11···O2^iii^	0.85 (3)	2.19 (3)	3.020 (3)	166 (4)
O1w—H12···O1^i^	0.85 (3)	2.55 (3)	3.137 (3)	128 (2)
N1—H1n···O1w	0.87 (3)	2.08 (3)	2.943 (4)	173 (4)
N1—H2n···O1^iv^	0.86 (3)	2.36 (3)	3.202 (4)	164 (3)

Symmetry codes: (i) *x*−1/2, −*y*+2, *z*; (ii) *x*−1/2, −*y*+1, *z*; (iii) −*x*+2, −*y*+1, *z*−1/2; (iv) −*x*+2, −*y*+2, *z*+1/2.
